# Deep Learning Approaches for the Assessment of Germinal Matrix Hemorrhage Using Neonatal Head Ultrasound

**DOI:** 10.3390/s24217052

**Published:** 2024-10-31

**Authors:** Nehad M. Ibrahim, Hadeel Alanize, Lara Alqahtani, Lama J. Alqahtani, Raghad Alabssi, Wadha Alsindi, Haila Alabssi, Afnan AlMuhanna, Hanadi Althani

**Affiliations:** 1 Departments of Computer Science, College of Computer Science and Information Technology, Imam Abdulrahman Bin Faisal University, Dammam 31451, Saudi Arabia2200002477@iau.edu.sa (L.A.);; 2 College of Medicine, Imam Abdulrahman Bin Faisal University, Dammam 31441, Saudi Arabia; 3 Department of Radiology, King Fahad University Hospital, Khobar 34445, Saudi Arabia

**Keywords:** germinal matrix hemorrhage (GMH), cranial ultrasound imaging, deep learning, YOLOv8 model, image classification, neonatal care

## Abstract

Germinal matrix hemorrhage (GMH) is a critical condition affecting premature infants, commonly diagnosed through cranial ultrasound imaging. This study presents an advanced deep learning approach for automated GMH grading using the YOLOv8 model. By analyzing a dataset of 586 infants, we classified ultrasound images into five distinct categories: Normal, Grade 1, Grade 2, Grade 3, and Grade 4. Utilizing transfer learning and data augmentation techniques, the YOLOv8 model achieved exceptional performance, with a mean average precision (mAP50) of 0.979 and a mAP50-95 of 0.724. These results indicate that the YOLOv8 model can significantly enhance the accuracy and efficiency of GMH diagnosis, providing a valuable tool to support radiologists in clinical settings.

## 1. Introduction

Germinal matrix hemorrhage (GMH) is a significant cause of morbidity in premature neonates, especially those with very low birth weight or gestational age less than 32 weeks [1,2]. This condition, characterized by bleeding within the fragile and vascularized germinal matrix, can progress to intraventricular hemorrhage (IVH), leading to severe neurological impairments such as cerebral palsy and cognitive disabilities [3,4]. Therefore, early detection and accurate grading of GMH are crucial for effective clinical management and better neurodevelopmental outcomes [5].

The conventional method of diagnosing GMH involves cranial ultrasound imaging, which relies on the subjective interpretation of experienced radiologists. This method, however, is prone to variability and can be time-consuming. Advances in deep learning have opened promising avenues for automating and enhancing diagnostic accuracy in medical imaging, particularly in conditions like GMH [6,7]. By integrating artificial intelligence with imaging diagnostics, it is possible to reduce variability and expedite diagnosis. In this study, we aim to develop and evaluate deep learning models for the automatic detection and classification of GMH in cranial ultrasound images.

We utilized a dataset from King Fahad Hospital that includes 586 neonates who underwent cranial ultrasound exams. Given the limited availability of public datasets on neonatal ultrasounds, privacy concerns, and the unique challenges of acquiring data from multiple hospitals, we were restricted to a single-source dataset. Although this dataset size is relatively small, it offers high-quality images and detailed labels, which are essential for accurate model training. Additionally, GMH is a rare condition, making this dataset uniquely valuable for research and clinical applications. To overcome the limitations of dataset size, we applied augmentation techniques and evaluated the performance of several deep learning models, including ResNet-18, ResNet-50, ResNet-152, and YOLOv8, for GMH detection and classification.

## 2. Related Work

### 2.1. Object Detection Method (YOLO) Studies

A study conducted by Qiao et al. in 2020 presented a sophisticated framework for automatically detecting cardiac chambers in fetal echocardiography images using an attention-based YOLOv4 model called HHRM-YOLOv4slim. This model incorporates a Multiscale Residual Hybrid Attention Module (MRHAM) with the CSPDarknet53 backbone to improve the learning of low-resolution images. The study tackled the issues posed by low-resolution images and the small size of fetal cardiac chambers, enhancing the model’s ability to identify robust and distinctive features. The HHRMYOLOv4-slim exhibited excellent performance, achieving a precision of 0.91, recall of 0.97, F1-score of 0.94, mean average precision (MAP) of 0.953, and detection speed of 43 frames per second (fps). This approach outperformed existing models, underscoring its potential to significantly improve the efficiency and accuracy of prenatal diagnoses of congenital heart diseases (CHDs) [8].

Also, in a study by Dadjouy and Sajedi in 2024, YOLO proved to be a powerful tool for object detection due to its speed and accuracy. Focusing on gallbladder cancer detection using ultrasound images, this research employed both YOLO and Faster R-CNN techniques to enhance detection performance. The dataset used, Gallbladder Cancer Ultrasound (GBCU), consisted of 1255 biliary ultrasound images categorized into malignant, benign, and normal cases. The methodology involved training the YOLOv8 model on this dataset, leveraging its efficiency in detecting accurately positioned bounding boxes. The results demonstrated that while YOLO achieved a true positive rate of 98.44%, combining YOLO with Faster R-CNN through a fusion method improved the mean Intersection over Union (mIoU) and overall classification accuracy, reaching 92.62%. This fusion method capitalized on YOLO’s precision in localization and Faster R-CNN’s accuracy in bounding box prediction, resulting in more reliable and accurate detection of gallbladder cancer [9].

Furthermore, a study titled “Detecting hemorrhage types and bounding box of hemorrhage by deep learning” focuses on using YOLOv4 to identify and categorize different intellectual hemorrhages, emphasizing the usage of bounding boxes to increase segmentation accuracy. The model was trained and validated using the Brain Hemorrhage Extended (BHX) dataset, which demonstrated superior precision, recall, and F1-scores for different types of hemorrhage. The results highlight the superiority of independent training methods over combined training approaches, with the former demonstrating higher metrics for recall and precision. The ability of YOLOv4 to withstand a variety of medical imaging problems, including changing image quality and the need for quick processing in emergency situations, further promoted its use. The use of YOLOv4 to detect intracranial hemorrhages represents a promising advancement in medical imaging, providing a quick, accurate, and efficient method for diagnosing neurologic conditions and neurology diagnostics. The application of cutting-edge deep learning architectures, such as YOLOv4, in the vital field of medical imaging has significantly helped this study, which has opened the door to improved automated medical diagnostics that may help in clinical decision-making, especially in areas with limited resources [10].

Moreover, Dadjouy and Sajedi et al. offered a thorough analysis of the existing literature on the use of AI in diagnosing gallbladder neoplasms on ultrasound images, emphasizing the major advances in diagnostic accuracy and efficiency that machine learning and deep learning approaches have brought about. This paper addresses the advantages and disadvantages of different AI techniques and suggests future research avenues to address outstanding issues. The study thoroughly evaluates several AI techniques, focusing especially on the use of the YOLOv8 model for object detection. The article demonstrated that testing a new fusion technique that integrated the bounding boxes from YOLOv8 and Faster R-CNN improved classification performance, achieving a noteworthy accuracy of 92.62%. With accuracy levels of 90.16% and 82.79%, respectively, the combined use of YOLOv8 and Faster R-CNN is a major improvement over this result. The study highlights how AI technologies have the ability to improve diagnostic accuracy while also facilitating the process to allow for more accurate and faster interventions [11].

The study conducted by Hao et al. in 2020 aimed to explore the use of the YOLO (You Only Look Once) architecture for locating the cerebellum in prenatal ultrasound scans. By utilizing the YOLO model, which is well-known for its efficacy in real-time object recognition, the study reveals excellent potential for speedy and precise medical diagnosis. The researchers achieved an average precision (AP) of 84.8% in the localization tasks on 78 test pictures by using a dataset of 316 ultrasound scans. The deep learning model was able to function well even with a small dataset due to the exceptionally successful implementation of transfer learning, which used pre-trained weights from the ImageNet database. This approach emphasizes the adaptation of universal deep learning models to specific, complicated medical imaging applications. The findings highlight deep learning technologies such as YOLO’s strong potential in medical imaging settings, especially in the challenging field of prenatal care, where prompt and precise diagnosis judgments are crucial. This study’s performance points to intriguing directions for future AI integration in diagnostic processes, which could improve the precision and effectiveness of prenatal screenings [12].

Another study by Yeditepe University researchers Burcu Selcuk and Tacha Serif in 2023 offers a thorough investigation into the use of the YOLOv8 algorithm for brain tumor detection utilizing MRI images. The study extends the use of the YOLOv8 model beyond generic object detection tasks by investigating modifications and optimizations made especially for the purpose of medical imaging. This involves adjusting the model to function well with MRI pictures, which have unique qualities and difficulties. The study intends to take advantage of the improved capabilities of YOLOv8 to precisely find and diagnose brain tumors, a crucial aspect in the treatment planning and prognosis of neurooncological conditions. It does this by using the BR35h dataset, which comprises of 800 annotated magnetic resonance images. The model’s excellent accuracy in identifying brain tumors is demonstrated by the study’s mean average precision (mAP) of 97.6%. A thorough examination is conducted on evaluation measures including precision, recall, and F1-score to give a comprehensive picture of the model’s performance during several testing and validation phases. When the model is being trained, additional information is obtained by looking at the loss values. Bounding box regression loss, object classification loss, and deformable convolutional network loss are discussed; these provide insight into the model’s learning process and its capacity to manage the fine features present in medical imaging tasks. The potential application of the YOLOv8 algorithm in clinical settings to improve brain tumor detection and medical care is highlighted in the paper’s conclusion. It emphasizes the model’s important contributions to raising the effectiveness of later medical interventions by enhancing the efficiency and accuracy of medical diagnostics [13].

In the next paper, authored in 2021 by Sudipto Paul, Dr. Md Taimur Ahad, and Md. Mahedi Hasan, the YOLOv5 model is utilized to advance the segmentation and detection of brain tumors from MRI images. The goal of this work is to apply advanced machine learning techniques to the field of medical imaging, namely neurology, where accurate and early diagnosis can have a significant impact on treatment strategies. The YOLOv5 method has been adapted to precisely address brain tumor segmentation. It is well-known for its rapid and precise object identification capabilities. With this adaptation, a portion of the 1992 brain MRI scans from the BRATS 2018 dataset were used to train and evaluate the model. This extensive dataset is essential because it offers a wide range of images, which are necessary for deep learning models to be trained on to function successfully in a variety of clinical cases. This model’s ability to segment brain tumors with an accuracy of 85.95% is a notable result of this research, showing its potential to optimize and improve brain tumor diagnostics. The effective use of YOLOv5 highlights the impact of AI in medical diagnostics to improve patient care outcomes with increases in accuracy and diagnostic speed [14].

In a study conducted by Wang et al. in 2024, the researchers aimed to develop an auxiliary diagnostic model using the YOLOv7 model architecture for classifying cervical lymphoma images, specifically differentiating between the benign nodes, lymphomas, and metastatic lymph nodes. The researchers used Ultrasonography images that were collected from the Zhangzhou Affiliated Hospital of Fujian Medical University, it included 2807 benign lymph nodes, 1108 lymphomas, and 4580 metastatic lymph nodes. Using augmentation, the dataset was expanded to 93,814 images and then divided into three sets: training (84,221 images), validation (4691 images), and testing (4902 images). Thereafter, the YOLOv7 was implemented and trained using the augmented dataset, along with the evaluation metrics including the mean average precision (mPA), accuracy, precision, recall, and F1-score. Moreover, the study results showed that the YOLOv7 model exceeded and outperformed radiologists at classifying cervical lymphoma. The model achieved an mAP of 96.4% at a 50% intersection-over-union threshold, with accuracy values of 0.962 for benign lymph nodes, 0.982 for lymphomas, and 0.960 for metastatic lymph nodes. Precision values for these categories were 0.928, 0.975, and 0.927. In contrast, the accuracy values achieved by the radiologists were significantly lower, at 0.659 for benign lymph nodes, 0.836 for lymphomas, and 0.580 for metastatic lymph nodes, with a corresponding precision value of 0.478, 0.329, and 0.596. Regardless of the great and promising results, the researchers faced some limitations in the retrospective nature and the use of data from a single institution, which can affect the generalizability of the results. Finally, the study results showed that the YOLOv7 model can enhance the accuracy of diagnosing cervical lymphadenopathy, which makes it a valuable tool for medical and clinical applications [15].

Another study conducted in 2023 by Kothala et al. aims to develop a YOLOv5x-GCB model to precisely detect the mixed intracranial hemorrhages in CT scans. The dataset used in this research contained 21,132 slices from 205 patients. The study was also supplemented by another online dataset from PhysioNet that contains segmentation data. The model integrated a ghost convolution to enhance the detection accuracy by reducing the computational complexity. The researchers trained the model using mosaic data augmentation, which enhanced the model resilience by creating different training samples, the evaluation metrics such as precision, recall, F1-score, and mean average precision (mPA). Moreover, the results of this study demonstrated that the model YOLOv5x-GCB exhibited an overall precision of 92.1%, recall of 88.9%, F1-score of 90%, and mAP pf 93.1%. These results show a significant performance compared to traditional models and other state-of-the-art deep learning methods, emphasizing the model’s ability to accurately detect several types of hemorrhage in a single CT scan. However, this study faced some limitations in the retrospective design and the inherent imbalance in the dataset used. But despite the limitation, the YOLOv5x-GCB model illustrates the advancement in the medical image analysis field, which offers a practical solution for the automatic detection and localization of mixed intracranial hemorrhage, and possibly improving diagnostic accuracy [16].

In 2024, a study conducted by Pham and Le et al. focused on developing a resilient model for detecting and classifying ovarian tumors in ultrasound images utilizing the YOLOv8 model architecture and focusing on three main configurations: detecting the presence of ovarian tumors, the classification of benign vs. malignant tumors, and the categorization into eight distinct tumor types. In this research, the dataset utilized was the OUT 2D-OS, which is a subset of the MMOTU dataset. It includes 1469 2D ultrasound images with expert annotations. The dataset was augmented to improve the model robustness and was divided into three sets: training, validation, and testing. Moreover, the researchers implemented the YOLOv8 model, as well as a state-of-the-art CNN model which is known for achieving high accuracy in object detection tasks; there were various versions of YOLOv8 (YOLOv8n, YOLOv8s, YOLOv8m, YOLOv8l, and YOLOv8) that were fine-tuned for these applications. The evaluation metrics in the study were precision, recall, and mean average precision (mPA). The results of the YOLOv8x outperformed all the procedures, resulting in a precision of 91.26%, a recall of 83.3%, and an mAP of 92.67% for detecting the presence of ovarian tumors task. The YOLOv8x attained an average precision of 71.5%, recall of 68.7%, and mAP of 70.6% for the classification of benign vs. malignant tumors task. As for the classification of benign and malignant tumor tasks, the YOLOv8x model achieved a precision of 74.9%, recall of 59%, and mAP of 66.8%. Despite promising results, the study faced some limitations, mainly the dataset and the imbalanced distribution of the tumor types posed. Moreover, the high rates of false positives and false negatives show that the model requires further refinement before it can be used in medical and clinical applications [17].

In 2024, a study was conducted by Lawrence Holland, which aims to improve the detection and classification of foreign bodies in ultrasound images by using AI segmentation models. Here, the researchers specifically compared the performance of YOLOv7 and U-Net models for identifying the shrapnel and the neurovascular features in ultrasound images. The utilized dataset consisted of 12,144 ultrasound images, along with the annotations for the shrapnel and neurovascular features. Here, the methodology of the study comprised pre-processing the images by resizing, normalization, and augmentation to enhance the model’s robustness and resilience. The two models were trained separately using pre-processed images. The two models were evaluated based on evaluation metrics such as precision, recall, intersection-over-union (IOU), and Dice coefficient metrics. In addition, a distance tri-age metric was developed to measure the proximity of shrapnel to neurovascular features, which was calculated using the segmentation masks generated by each model. Moreover, the study results showed that U-Net achieved over 90% validation accuracy in the first epoch, while YOLOv7 achieved a validation precision of 82.2% in nine training epochs. As for the single-class shrapnel segmentation, both models performed similarly with an accuracy of 99.1%. However, YOLOv7 achieved a higher precision of 76% compared to U-Net which achieved a precision of 63.8%, while U-Net had a higher recall of 67.7% than YOLOv7, which achieved 74.3%. As for the multi-class segmentation, YOLOv7 had a precision of 84.8% and recall of 84.2%, while U-Net achieved a final accuracy of 96.5% after twenty training epochs. YOLOv7 produced more precise prediction masks, whereas U-Net’s masks were generally larger, resulting in higher recall but more false positives. Furthermore, the study faced some limitations, including the limited size of the utilized dataset, which affected the model’s generalizability. Future work suggests expanding the dataset, including multiple labelers, and validating the models with in vivo medical images to enhance clinical abilities. In conclusion, both models are efficient and effective for segmenting foreign bodies in ultrasound images in ultrasound images [18].

In 2024, a study by Widayani et al. explored the use of the YOLOv8 DL algorithm for several medical imaging tasks, such as organ segmentation, disease classification, and lesion detection. This study systematically reviewed the performance of YOLOv8 across different imaging modalities, including X-ray, CT-Scan, and MRI. Results showed high precision (over 0.92), recall (over 0.90), and mean average precision (mAP over 0.93) in detecting meningiomas and pituitary tumors. However, glioma detection showed relatively lower performance. In breast cancer detection using a stochastic gradient descent (SGD) optimizer, YOLOv8 achieved an average mAP of 0.87. The model also performed well in dental radiography, detecting cavities, impacted teeth, fillings, and implants with precision above 0.82. For lung disease classification, YOLOv8 achieved 99.8% accuracy in training and 90% in validation, demonstrating its effectiveness in medical imaging applications and its potential to enhance clinical decision-making and patient outcomes [19].

Another study by Inui et al. in 2023 used YOLOv8 to identify elbow osteochondritis dissecans (OCD) in ultrasound pictures. The study included 2430 images classified into normal and OCD lesions. The YOLOv8 model has achieved a high diagnostic accuracy, with an accuracy of 99.8%, recall of 99.75%, precision of 100%, and F-measure of 99.87%. The mean average precision (mAP) for the bounding box detection was 0.994 and 0.995 for YOLOv8n and YOLOv8m models, respectively. This study emphasizes the model’s potential for early OCD detection in youth baseball players, which is critical for effective conservative treatment and prevention of disease progression [20].

Moreover, in 2023, a comprehensive review by Passa et al. investigated the application of the YOLOv8 algorithm for MRI brain tumor detection. The study utilized data augmentation techniques to improve the performance of the YOLOv8 model in identifying meningiomas, gliomas, and pituitary tumors. The study demonstrated that data augmentation significantly enhanced detection accuracy, with mAP values reaching 0.986 for meningiomas, 0.894 for gliomas, and 0.975 for pituitary tumors, which underscores the importance of data preprocessing and augmentation in optimizing deep learning models for medical imaging [21].

Furthermore, in 2024, a study by Qureshi et al. reviewed YOLO-based object detection in medical imaging from 2018 to 2023. The review covered several applications, including breast cancer detection, lung nodule detection, and polyp detection in colonoscopy images. The study found that YOLO models, specifically the latest versions, achieved high detection accuracy and efficiency. For instance, the YOLO-V5 model, enhanced with a self-attention mechanism, significantly improved polyp detection accuracy. The review highlighted the evolution of YOLO models in terms of structure and training techniques, contributing to their success in real time and accurate medical image analysis [22].

### 2.2. Deep Learning Studies

A research study by Yan et al. in 2024 introduced a deep learning approach for segmenting the pulmonary artery (PA), aorta (Aoa), and superior vena cava (SVC) in fetal heart ultra-sound images with the aim of prenatal screening for congenital heart diseases (CHDs). This approach employs a two-step process using a YOLOv5 model for initial detection, followed by DeepLabv3, which features an attention-based multi-scale feature fusion (AFMF) module for precise segmentation. The YOLOv5 model is essential for accurately identifying regions of interest (ROIs), which are then finely segmented by the AFMF-DeepLabv3 model. Testing this method on a dataset of 58 fetal heart 3VV images showed that the YOLOv5 model, in conjunction with DeepLabv3, markedly enhanced the segmentation accuracy for PA, Aoa, and SVC to 85.5%, 89.1%, and 77.5%, respectively. This highlights the effectiveness of YOLOv5 in improving the precision and efficiency of prenatal screening for CHD through reliable and automated fetal cardiac ultrasound analysis [23].

Additionally, in 2024, Natali et al.’s study introduces a deep learning model for real-time prostate detection in transabdominal ultrasound (TAUS) images. The proposed detection transformer model, NanoDeTr, is compared with the YOLOv8 model. The research aims to assist inexpert operators by providing an automatic detection system that reduces patient discomfort associated with transrectal procedures. The models were trained and evaluated on a dataset of 7500 ultrasound frames, achieving a mean average precision at 0.50 (mAP@50) of 0.95 for NanoDeTr and 0.98 for YOLOv8, indicating high detection accuracy. The YOLOv8 model demonstrated slightly better performance in terms of precision and recall, while NanoDeTr excelled in running speed with 993 queries per second (QPS). This study highlights the potential of using advanced deep learning models to enhance the efficiency and accuracy of prostate cancer detection in clinical settings [24].

The main goal of a study conducted in 2023 by Basak et al. is to develop a sophisticated deep learning model that can efficiently detect and then classify the different types of Intracranial Hematoma in CT images. This study used a public online dataset named Brain Hemorrhage Extended (BHX), which consists of 15,921 images with 39,668 bounding box annotations for the six subtypes of hematoma, which are intraparenchymal hemorrhage (IPH), intraventricular hemorrhage (IVH), subdural hemorrhage (SDH), subarachnoid hemorrhage (SAH), epidural hemorrhage (EDH), and chronic subdural hemorrhage (CHR). The proposed method in this study includes several improvements to the YOLOv5 model architecture, such as adding a window-based stacking approach for pre-processing the CT scans and a Cascade Attention Model (CAM) to enhance the feature extraction process. This study employs a combination of a single and stacked window setting to detect the hematoma regions effectively, with the stacked window approach. Moreover, the model performance was evaluated using different metrics such as precision, recall, F1-score, mAP@0.5, and mAP@0.5:0.95 of 0.935, 0.908, 0.921, 0.943, and 0.65, respectively. These results indicate significant improvements over previous models, particularly in detecting multiple hematoma sub-types in a single CT image and distinguishing between acute and chronic cases. However, the researchers have emphasized several limitations, one being the difficulties the model faced in accurately detecting the hematomas that overlap with the tissues and the cross-multi-classification between the similar appearance of hematoma sub-types. In addition, the utilized dataset has fewer positive instances for some of the hematoma subtypes, which may limit the model’s generalizability. In conclusion, future work suggests addressing the issues by including new feature fusion techniques and training the model on other open-source datasets to enhance the model’s resilience [25].

Another study in 2023, conducted by Cortes-Ferre et al., investigated the application of deep learning, specifically EfficientDet and Grad-CAM, for the detection of intracranial hemorrhage (ICH) in CT scans. The study achieved a classification accuracy of 92.7% and an area under the ROC curve of 0.978. The proposed model not only classified the presence of hemorrhage but also provided visual explanations of its predictions, enhancing its utility as a decision-support system in clinical settings. This research highlights the potential of integrating deep learning with medical imaging to improve diagnostic accuracy and workflow efficiency in detecting critical conditions like ICH [26].

In 2021, the study explored the application of a novel deep learning-based model for diagnosing intracranial hemorrhage (ICH). The dataset used comprises CT scans from 82 individuals, aged up to 72 years, containing images across six classes: Intraventricular, Intra-parenchymal, Subdural, Subarachnoid, Epidural, and No Hemorrhage. The model involves preprocessing using Kapur’s thresholding with the Elephant Herd Optimization (EHO) algorithm for segmentation, feature extraction through the Inception v4 network, and classification using a multilayer perceptron (MLP). Results from the study indicate high performance with the model achieving a sensitivity of 94.52%, specificity of 96.34%, precision of 96.10%, and accuracy of 96.03% at 500 epochs. This study significantly contributes to enhancing ICH diagnosis by integrating effective segmentation and deep learning architectures [27].

### 2.3. Machine Learning Studies

Lin et al.’s study, which was conducted in 2023, focused on creating a machine learning model designed to detect congenital abnormalities in the central nervous system (CNS) using fetal cerebral-cranial ultrasound scans and evaluate its ability to enhance diagnostic accuracy among clinical practitioners. The AI model, built on YOLOv3, was trained with 37,450 images from 15,264 fetuses and tested with 18,920 images from 4496 fetuses, achieving an overall accuracy of 79.1%, sensitivity of 78.4%, specificity of 94.4%, and an area under the curve AUC of 86.5%. The AI system performed on par with expert doctors. Furthermore, when used by trained doctors, the AI system significantly improved their diagnostic accuracy and performance scores. This study highlights the potential of AI to aid less experienced physicians in prenatal diagnostics, thus enhancing the detection rates of fetal CNS malformations [28].

In a 2019 study published in the European Journal of Pediatrics, researchers conducted a respective cohort analysis on extremely preterm infants (28 weeks of gestation) at a tertiary NICU in Australia. The study compared cranial ultrasound (cUS) and magnetic resonance imaging (MRI) for detecting brain injuries and correlating these with neurodevelopmental outcomes at 1 and 3 years. The dataset included general patient information and neurodevelopmental scores. The study found that severe abnormalities detected by TEA-cUS were reliable predictors of adverse outcomes, whereas MRI, despite detecting more subtle abnormalities, had poor predictive value for long-term impairment. This suggests that routine MRI may not be necessary for all preterm infants, highlighting cUS as a cost-effective and accessible primary imaging modality. The findings underscore the need for ongoing assessments and early interventions for high-risk preterm infants to improve long-term outcomes [29].

In 2019, a study developed a novel framework for automated and accurate intracranial hemorrhage (ICH) detection using a combination of convolutional neural networks (CNNs) and recurrent neural networks (RNNs). The dataset included 2836 subjects from three hospitals, categorized into five ICH subtypes. CNN extracted features from image slices while RNN captured sequential information, resulting in robust classification performance. The framework provided two training scenarios: subject-level and slice-level annotations. Evaluation against two groups of head CT interpreters demonstrated the model’s potential, contributing significantly to the field of ICH diagnosis [30].

## 3. Materials and Methods

### 3.1. System Framework

**Figure 1:**
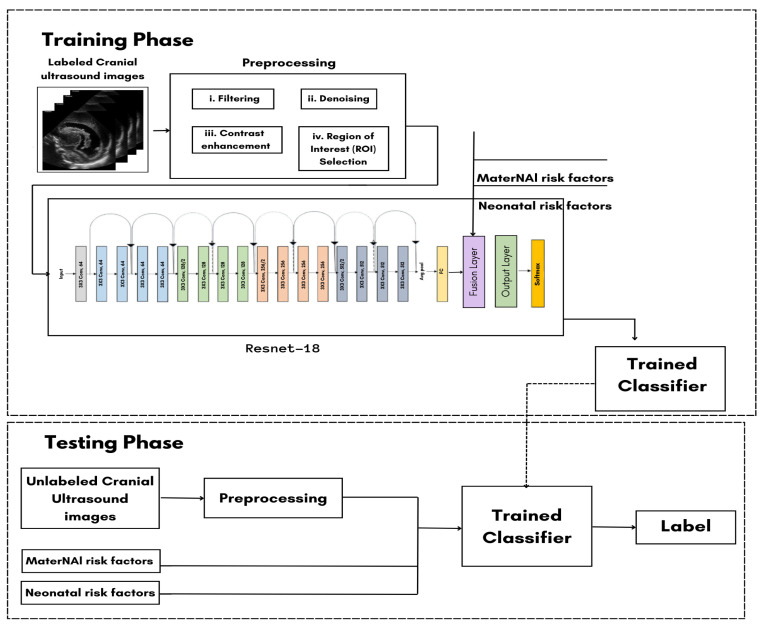
Classification training model.

As shown in Figure 1, the system requires the user to input the necessary data, which includes labeled cranial ultrasound images and MaterNAI/Neonatal risk factors. The process begins with preprocessing the ultrasound images through steps like filtering, denoising, contrast enhancement, and region of interest (ROI) selection.

The preprocessed images then enter the ResNet-18 model, where the image data are fused with the text input (MaterNAI and neonatal risk factors) at the late fusion layer. This fusion results in the combination of both visual and textual data, which is crucial for generating accurate predictions. During the training phase, 70% of the dataset is used for training the model. The preprocessed images and text inputs pass through the ResNet-18 model, and the results are saved in the trained classifier. For the testing phase, the remaining 30% of the dataset undergoes the same preprocessing steps and passes through the trained classifier. The system compares the testing results to the training data to calculate the model’s accuracy. Ultimately, this process will support the detection, grading, and standardization of diagnoses for GMH.

### 3.2. Study Population

**Figure 2:**
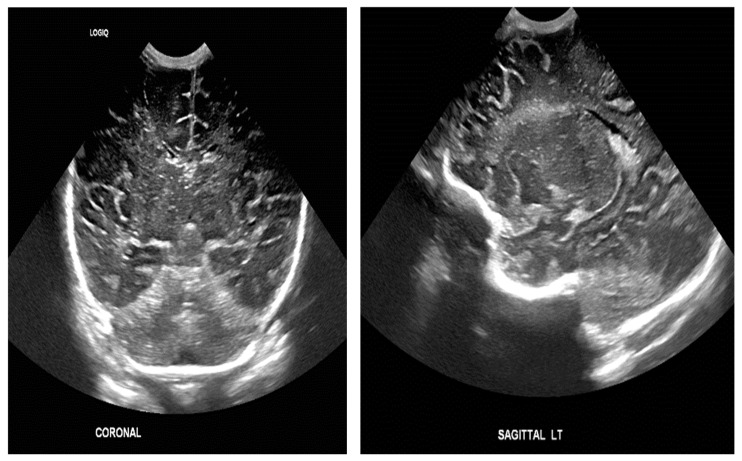
Sample of the dataset ((**left**): coronal US, (**right**): left US).

The study population comprised neonates who underwent cranial ultrasound (US) at King Fahad Hospital of the University (KFUH) in Khobar, Kingdom of Saudi Arabia. Neonatal cerebral ultrasound images were collected for this study. The inclusion criteria encompassed neonates who had undergone cranial US at KFUH, with complete sets of left sagittal, right sagittal, and coronal cranial ultrasound images, and those with confirmed diagnoses of germinal matrix hemorrhage (GMH), as shown in Figure 2. Exclusion criteria included neonates without complete sets of the required cranial US images and patients with incomplete or missing medical records. The study initially considered a total of 582 neonates who underwent cranial US, among whom approximately 40 were identified to have GMH.

### 3.3. Data Collection

We reviewed a hospital database of 586 neonates who underwent cranial ultrasound examinations over five years, focusing on germinal matrix hemorrhage (GMH). Due to ethical considerations and the rarity of this condition, data availability was limited. Our inclusion criteria prioritized high-quality images from advanced ultrasound devices, excluding those from portable devices due to noise concerns. Although this decision reduced the dataset size, it ensured data reliability and consistency, crucial for training accurate deep learning models.

Despite the smaller dataset, our model benefits from the detailed and high-quality labels. Each neonate’s brain regions (sagittal left, sagittal right, and coronal views) were comprehensively covered, and bilateral GMH cases were classified separately to capture the full spectrum of severity. This focus on a rare and critical condition adds significant value to the dataset.

While larger datasets are generally preferred for deep learning, we addressed the limitations of our smaller dataset through advanced augmentation techniques, which artificially increased the diversity of the training data. This approach allows our model to generalize effectively across the different GMH grades, even for rare cases like Grade 4 hemorrhages. Additionally, our dataset’s focus on rare and severe conditions highlights its clinical significance, as early detection and accurate classification of GMH can have profound implications for patient care and treatment decisions.

In future iterations, we aim to explore advanced noise reduction and image enhancement techniques to incorporate images from portable devices and further expand the dataset, balancing data quantity with quality. However, the dataset we have curated remains a significant contribution to GMH research, given the rarity of such high-quality, labeled medical data in neonatal care.

The dataset was meticulously organized into patient-specific folders, each labeled with a unique patient ID and containing the corresponding ultrasound images. This systematic approach to data management facilitated efficient retrieval and analysis of the images.

### 3.4. Dataset Augmentation and Preprocessing

To optimize the performance of the YOLOv5 model for detecting germinal matrix hemorrhages (GMHs), we implemented several preprocessing and augmentation techniques aimed at enhancing data quality and increasing the dataset’s diversity. Given the relatively small size and imbalance of our dataset, these steps were crucial for improving model generalization and mitigating the risks of overfitting.

**Figure 3:**
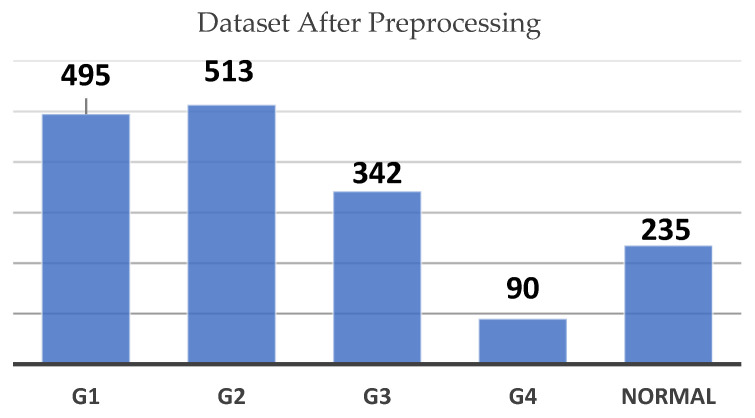
Dataset count by grade before preprocessing chart.

Initially, the dataset was highly imbalanced, as shown in Figure 3. G1 (Grade 1) contained the largest number of images at 60, followed by G2 (Grade 2) with 57, and the Normal class with 47 images. G3 (Grade 3) held 41 images, while G4 (Grade 4) was significantly underrepresented, with only 7 images. This imbalance posed challenges for training a deep learning model, as underrepresented classes like G4 could lead to poor model performance in those categories.

**Figure 4:**
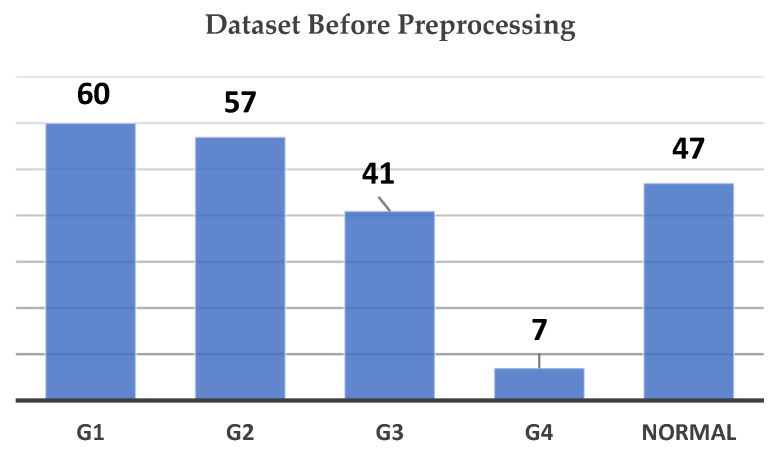
Dataset count by grade after preprocessing chart.

To mitigate this issue, we utilized a range of data augmentation techniques. These included horizontal and vertical translations (±10%), zooming in and out (±5%), rotations (±10°) as shown in Figure 4, and random horizontal flips. These augmentations simulated variations that could occur during real-world image acquisition, such as shifts in scan angles or changes in focus, making the model more robust in recognizing hemorrhagic regions under diverse conditions.

So, augmentation was performed to artificially expand the number of images in the underrepresented classes. After augmentation, the dataset saw a significant increase in images for each class, as illustrated in Figure 4. G2 now contains 513 images, G1 has 495, and the Normal class includes 235 images. G3 has increased to 342 images, while G4 has risen from 7 to 90 images.

While these augmentation techniques helped address the imbalance, we recognize that augmentation alone may not completely resolve the challenge posed by such limited examples in classes like G4. In future work, we aim to explore more advanced methods, such as class reweighting, synthetic data generation, or transfer learning from larger, similar datasets, to further enhance the model’s ability to generalize across all classes.

**Figure 5:**
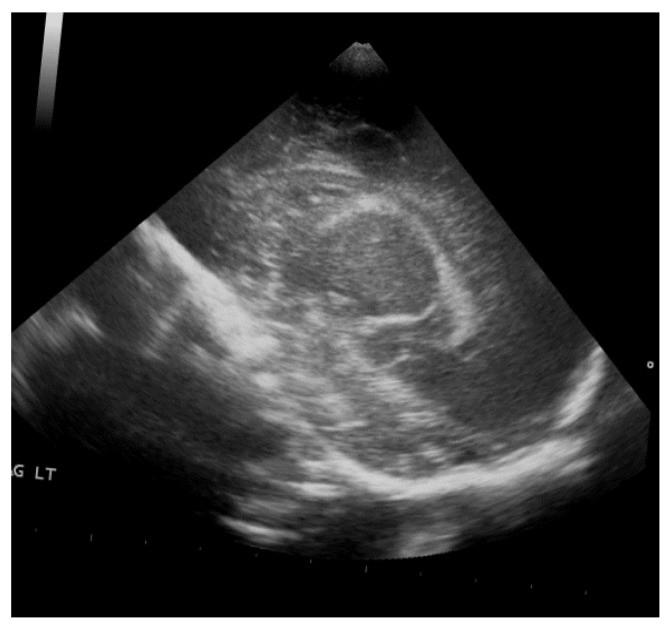
Rotation by 10%.

To address preprocessing, we applied cropping to automatically center and focus on the region of interest (ROI) in each image, as shown in Figure 5. This step removed irrelevant background and emphasized the critical areas where hemorrhages were most likely to appear. We followed this by resizing all images to a uniform size of 255 × 255 pixels to ensure consistency across the dataset, allowing the model to process the images efficiently while maintaining sufficient resolution for identifying fine details in the ultrasound scans.

**Figure 6:**
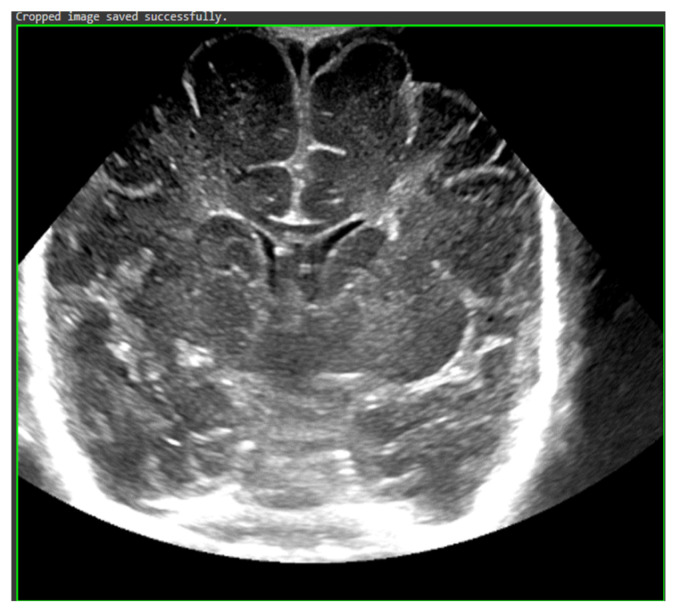
Cropped ROI.

Accurate annotation was performed using Roboflow, with bounding boxes manually drawn around hemorrhagic areas under the guidance and supervision of KFHU doctors. Different colors were assigned to represent each hemorrhage grade (g1, g2, g3, g4) and the Normal class, ensuring the model could effectively distinguish between them. This meticulous annotation process allowed the YOLOv8 model to focus on the most relevant areas, significantly enhancing its functionality. For instance, Figure 6 illustrates an orange box marking a Grade 3 hemorrhage, a red box for Grade 4, a purple box for Grade 1, a green box for Grade 2, and a yellow-green box for Normal class. Together, these preprocessing and augmentation techniques played a critical role in enhancing the quality and diversity of the dataset, which in turn improved the model’s performance in detecting and classifying GMH across various grades.

### 3.5. Conducted Experiments

In this study, we conducted three experiments using ResNet-18, ResNet-50, and ResNet-152 models. Comparing these experiments revealed extensive differences in performance and suitability for GMH detection and classification.

**First Experiment using Resnet-18:** The first experiment utilized the Residual Network with 18 deep layers (ResNet-18), a well-known model widely used for classification and object detection. We loaded the pre-trained ResNet-18 model from the TorchVision library, which consisted of 20 convolutional layers with filter sizes ranging from 64 to 512, one max pooling layer, and three downsampling layers. We trained the model on the preprocessed dataset, randomly splitting the data into 80% for training and 20% for testing. The results from this model, illustrated in Table 1, showed promising accuracy in initial tests. However, when evaluated on the entire dataset, it failed to maintain consistent performance.

**Second Experiment using ResNet-50:** The second experiment was conducted using the Residual Network with 50 deep layers (ResNet-50). We loaded the pre-trained ResNet-50 model from the TensorFlow library, which consists of 53 convolutional layers with filter sizes ranging from 64 to 2048, along with one max pooling layer. In this experiment, we divided the dataset into three segments based on the imaging sides: left, right, and coronal. Each subset comprised five classes: Grade 1, Grade 2, Grade 3, Grade 4, and Normal. Despite careful segmentation, the model struggled significantly with imbalanced data, particularly for underrepresented classes such as Grade 4, yielding deficient results, as demonstrated in Table 2.

**Figure 7:**
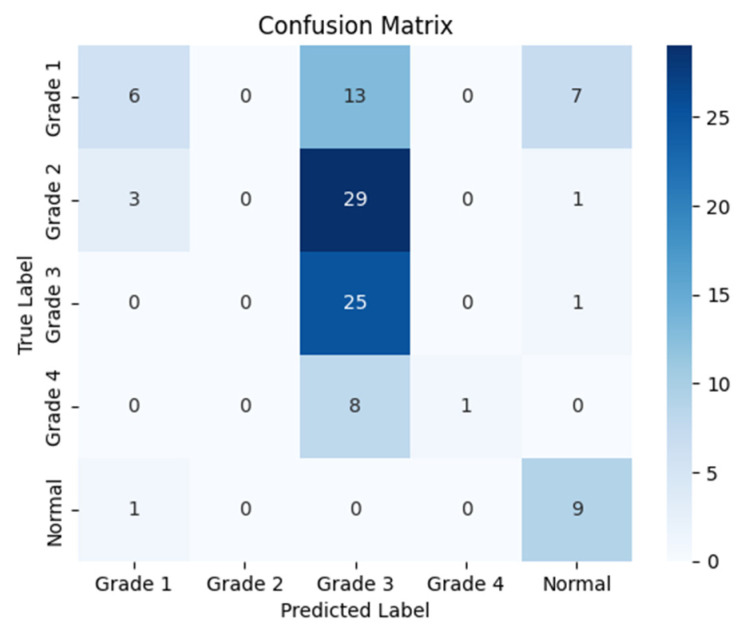
Confusion matrix for the right side for Resnet-50.

**Figure 8:**
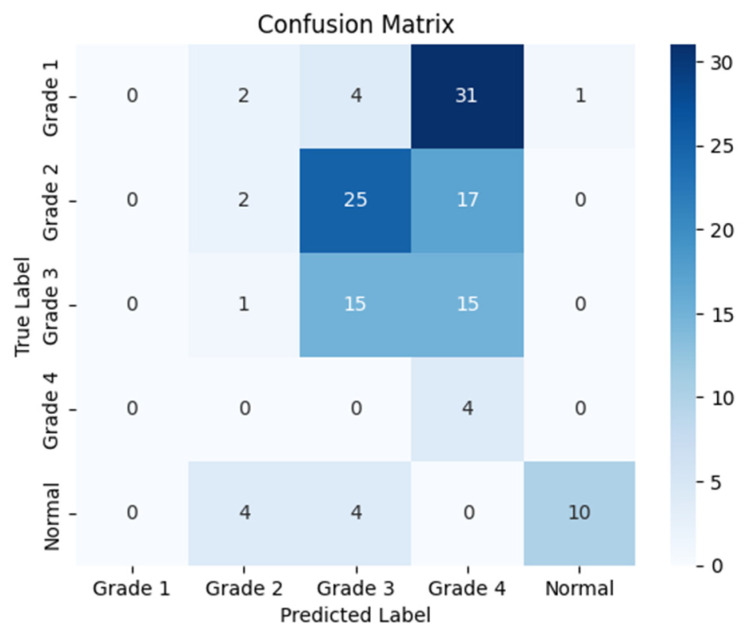
Confusion matrix for the left side for Resnet-50.

**Figure 9:**
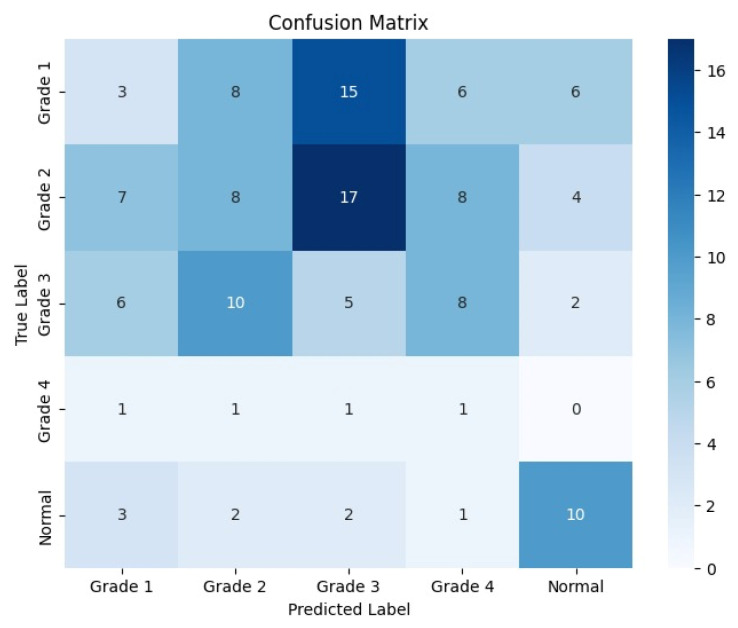
Confusion matrix for the coronal side for Resnet-50.

The following confusion matrices in Figure 7, Figure 8 and Figure 9 illustrate the classification performance of each model, providing insights into false positives and false negatives that may have significant clinical implications, as follows:

**Third Experiment using Resnet-152:** The third experiment was conducted using the Residual Network with 152 deep layers (ResNet-152), designed primarily for recognition and classification tasks. We loaded the pre-trained ResNet-152 model from the TensorFlow libraries. This model comprises 151 convolutional layers (including the initial layers) with filter sizes ranging from 64 to 2048, one max pooling layer, and two dropout layers (the first with a rate of 0.5 after the dense layer with 1024 units, and the second with a rate of 0.3 after the second dense layer with 512 units). Following the same approach as the second experiment, we trained this model on the provided dataset, which was randomly split into 80% for training and 20% for testing. Although this model showed improved performance on coronal images, it performed inadequately for the left and right sides, as illustrated in Table 3.

**Figure 10:**
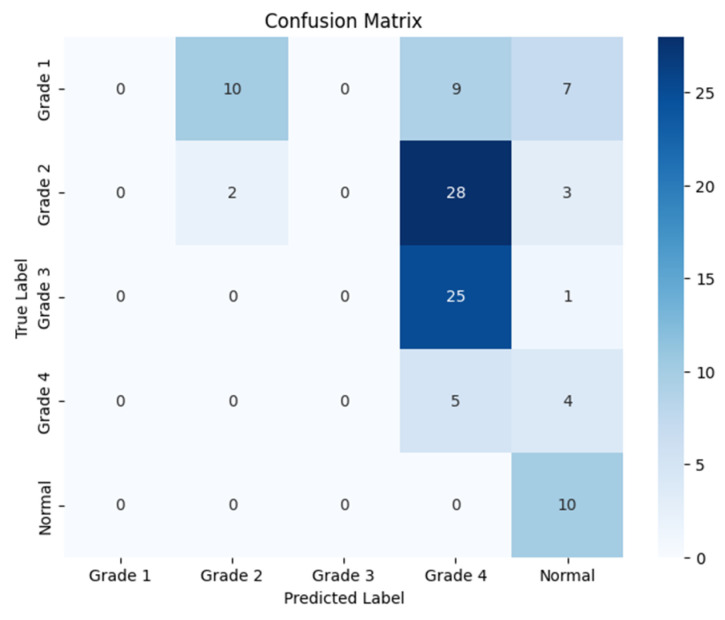
Confusion matrix for the right side for Resnet-152.

**Figure 11:**
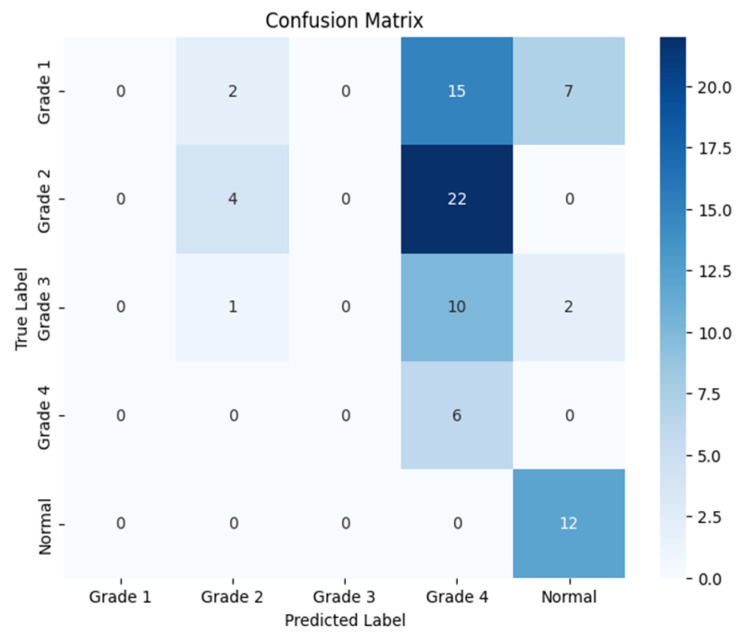
Confusion matrix for the left side for Resnet-152.

**Figure 12:**
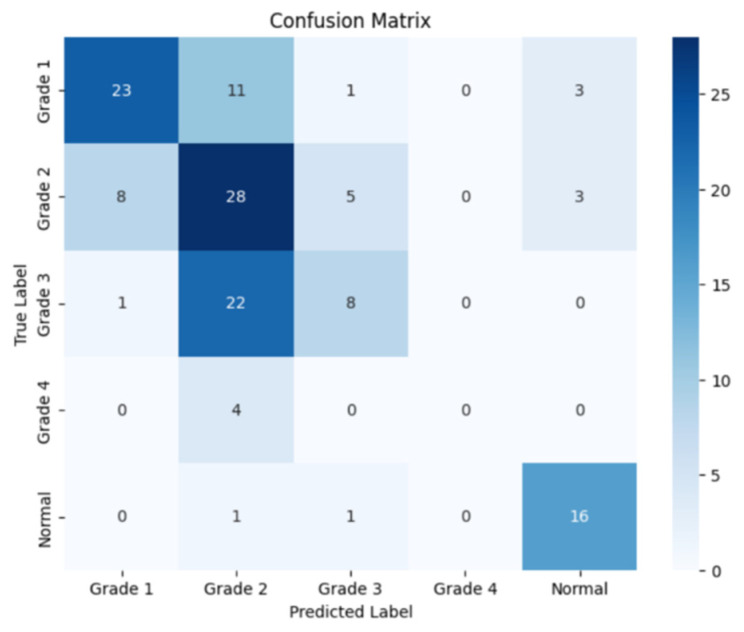
Confusion matrix for the coronal side for Resnet-152.

The following confusion matrices in Figure 10, Figure 11 and Figure 12 illustrate the classification performance of each model, providing insights into false positives and false negatives that may have significant clinical implications, as follows:

The following Table 4 summarizes the accuracy results for each model (ResNet-18, ResNet-50, and ResNet-152) across the three imaging segments: left, right, and coronal views. These results highlight the differences in the performance of the models based on the side from which the cranial ultrasound images were taken. As shown, ResNet-18 consistently outperformed the other models, particularly in the left view, while ResNet-50 and ResNet-152 demonstrated significant challenges, especially in the right view.

### 3.6. Model Training

**Figure 13:**
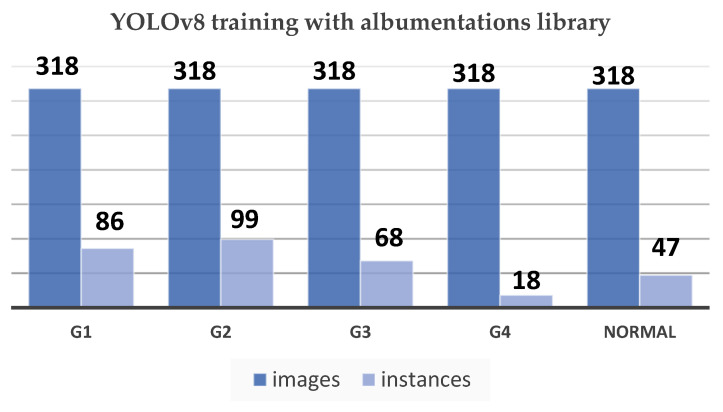
YOLOv8 training with Albumentations library chart.

After noticing no positive changes in the previous experiments, we decided to change the model as well as our approach; this time we used the YOLOv8 (You Only Look Once) model by utilizing the ultralytics library [31]. Training this model involves several primary steps to achieve optimal performance. As shown in Figure 13, the data are organized into five classes: Grade 1, Grade 2, Grade 3, Grade 4, and Normal. By benefiting from transfer learning, the pretrained weights were imported in order to adapt the model, especially for this task.

**Figure 14:**
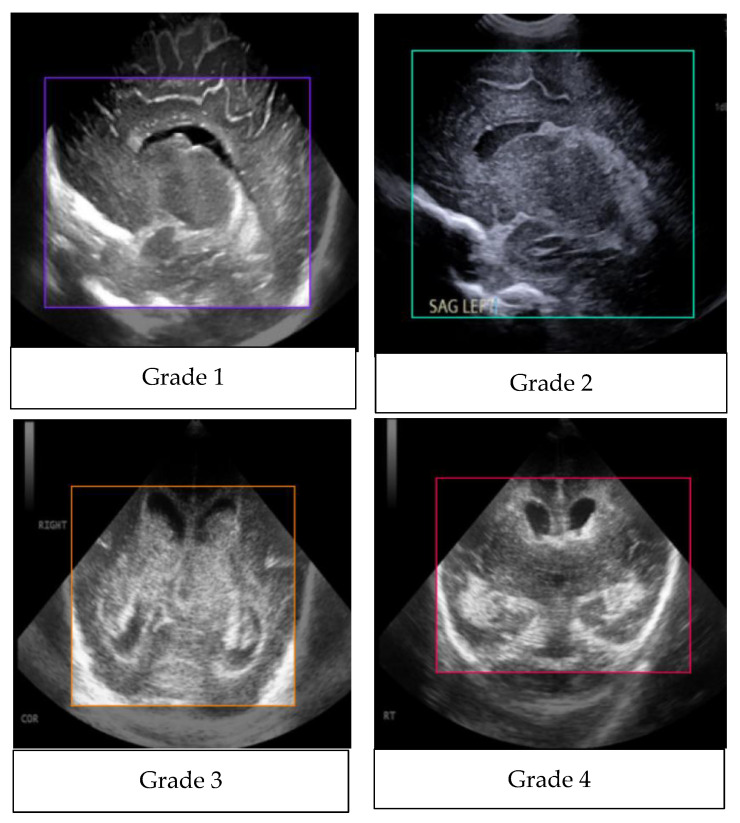
Annotated images.

The YOLOv8 model relies heavily on annotation, which establishes the foundation for successfully training the algorithm to identify and categorize objects within images. This method was made possible using third-party applications like Roboflow, which made it possible to efficiently label a number of classes, including g1, g2, g3, g4, and Normal. This method improves the model’s ability to detect various abnormalities in medical images, especially when it comes to detecting germinal matrix hemorrhages. By means of careful annotation, the model receives the necessary labeled data to identify and extrapolate patterns, which allows for accurate hemorrhagic region detection and categorization. By drawing boxes above the hemorrhagic white areas, the annotation method makes sure the model concentrates on important information, which improves inference accuracy. Therefore, this thorough annotation approach significantly improves the YOLOv8 model’s functionality. As shown in Figure 14, there is the purple box for Grade 1, green box for Grade 2, orange box for Grade 3, red for Grade 4, and yellow-green for Normal.

**Figure 15:**
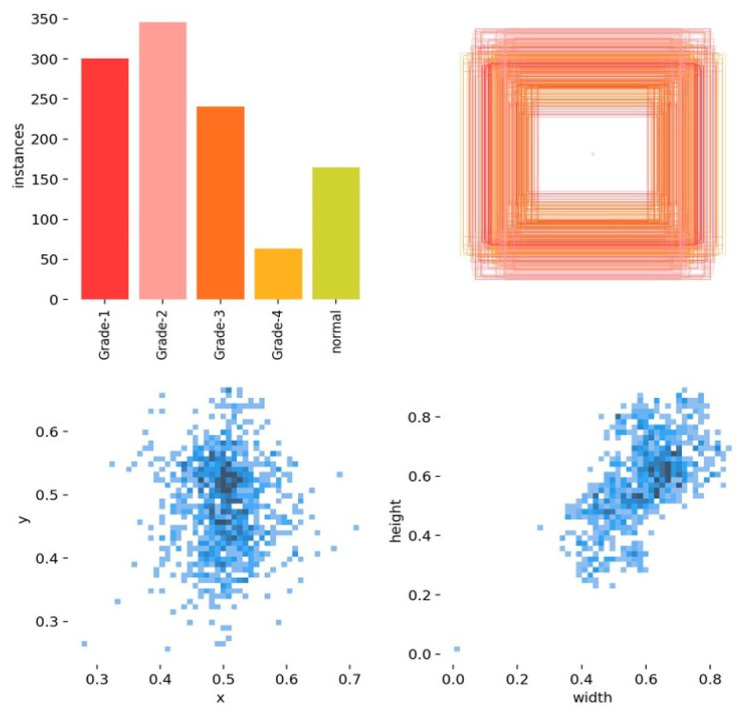
An analysis of the bounding box annotations.

Figure 15 shows an analysis of the bounding box annotations in the GMH classification with observations of the label analysis, as follows:

- Grade 2 has the most instances.
- Grade 4 has the least instances.
- The annotations are concentrated in the central area of the images, indicating a specific region of interest.
- There is a positive correlation between bounding box width and height.

The YOLOv8 model architecture, which consists of 225 layers and about 11.1 million parameters, was adjusted to recognize and comprehend these five classes. The training of the model was running on 100 epochs along with eight data loader workers, while the Tensor Board logs and visualizes the training process in real time and tracks the evaluation metrics. Moreover, the training environment for the model was set up to manage the computational requirements of YOLOv8. Using Google Collab Pro has provided us access to a T4 GPU and high RAM setting, which optimized efficiency. There are some necessary Python Ver 3.10 tools and libraries such as ultralytics, roboflow, OpenCV-python, torch, torch vision, and finally pytorch-lighting, which were imported and used to manage the dataset, build the model, and for visualization. Also, this process includes augmentations from the Albumentations library to enhance the model’s resilience. In addition, the training process for the model includes splitting the dataset into training, validation, and testing subsets in a ratio of 70–20–10. Here, the pre-processing step ensures that all the images are resized to a unified size which is 640 × 640 pixels before being inputted into the model. Also, an adaptive optimizer is used to automatically decide the optimal learning rate and momentum. During the training process, evaluation metrics such as the F1–Confidence Curve, Precision–Confidence Curve, and Precision–Recall Curve will be monitored to assess and analyze the performance of the model. A thorough analysis of these metrics assists in fine-tuning the model and handling challenges, such as distinguishing between similar grades such as Grade 2 and Grade 3, this will guarantee a strong and resilient classification model for GMH.

### 3.7. Model Performance

The performance of the finished model was evaluated by testing the data. Confusion matrices were constructed using the scikit-learn library, which provided perceptions into the methodology of YOLOv8 model. The YOLO loss function is composite, designed to train the model on several tasks simultaneously: accurately predicting bounding box parameters and class probabilities. The loss function consists of several components, as follows:

$$\lambda_{coord\;\;\;\;}\overset{S^{2}}{\underset{i=0}{\sum }}\;\overset{B}{\underset{j=0}{\sum }}1_{ij}^{\mathrm{obj}}\;\left[{\left(x_{i}-{\hat{x}}_{i}\right)}^{2}+{\left(y_{i}-{\hat{y}}_{i}\right)}^{2}\;\right]\;+\;\lambda_{coord\;\;\;\;}\overset{S^{2}}{\underset{i=0}{\sum }}\;\overset{B}{\underset{j=0}{\sum }}1_{ij}^{\mathrm{obj}}\;\left[{\left(\sqrt{w_{i}}-\sqrt{{\hat{w}}_{i}}\right)}^{2}+{\left(\sqrt{h_{i}}-\sqrt{{\hat{h}}_{i}}\right)}^{2}\;\right]\;+\;\overset{S^{2}}{\underset{i=0}{\sum }}\;\overset{B}{\underset{j=0}{\sum }}1_{ij}^{\mathrm{obj}}\;\;{\left(C_{i}-{\hat{C}}_{i}\right)}^{2}\;+\;\lambda_{\mathrm{noobj}\;\;\;\;}\overset{S^{2}}{\underset{i=0}{\sum }}\;\overset{B}{\underset{j=0}{\sum }}1_{ij}^{\mathrm{noobj}}\;{\left(C_{i}-{\hat{C}}_{i}\right)}^{2}\;+\;\overset{S^{2}}{\underset{i=0}{\sum }}1_{i}^{\mathrm{obj}}\;\;\underset{c\in \mathrm{classes}}{\sum }{\left(p_{i}\left(c\right)-{\hat{p}}_{i}\left(c\right)\right)}^{2}$$

**Coordinate Loss:** Penalizes deviations from the true bounding box (box center coordinates b_(x), b_(y), width b_(w), and height b_h). It includes terms for the box’s center, width, and height differences, weighted by λ_coord to emphasize the importance of accurate localization.

**Class Probability Loss:** Penalizes the difference in the predicted probabilities for the classes, ensuring the predicted class matches the true class.

Each bounding box prediction consists of five elements: b_(x), b_(y), b_(w), b_h, and p_c (the confidence score).

The YOLO algorithm is a cornerstone in real-time object detection due to its efficiency and accuracy, allowing it to run well even on limited hardware [32].

## 4. Results and Visualization

The YOLOv8 model illustrated excellent results in classifying the images into five categories. Throughout the validation process, the model demonstrated its capabilities in accurately differentiating between the given classes, as well as achieving high precision and recall scores over the board. The validation metrics shown in Table 5 were remarkable, the YOLOv8 model maintained a mean average precision (mAP50) of 0.979, which reflects its efficiency in accurately recognizing and detecting objects in the input images. As for the mAP50-95, which explains the range of intersection over union (IoU) thresholds, it was 0.724, which highlights the model’s robust performance. Furthermore, a comprehensive analysis of the Precision–Confidence and Recall–Confidence curves denotes that the YOLOv8 model presented a good and consistent performance even at varying confidence thresholds, ensuring reliable and resilient predictions. Moreover, the model’s capabilities of handling data augmentation and pre-processing efficiently played an essential key in its success. By resizing the input images into a unified size of 640 × 640 pixels and applying multiple augmentation methods, the model was able to generalize better and become more robust to dataset variability. This step was essential, considering the imbalanced nature of the used dataset, where some classes are underrepresented. Additionally, the confusion matrices that were constructed over the validation phase presented a detailed view of the model’s performance, emphasizing its strength in detecting “Normal” and “Grade 4” classes with almost perfect precision and recall. Nevertheless, the confusion matrices showed a small challenge in distinguishing between Grade 2 and Grade 3, since they have close bleeding placements, which highlights the inherited complications in the medical image classifications. In general, the YOLOv8 model demonstrated its ability to be an effective and reliable potential tool that can be used in GMH detection and classification, capable of assisting radiologists in making an accurate diagnosis.

**Figure 16:**
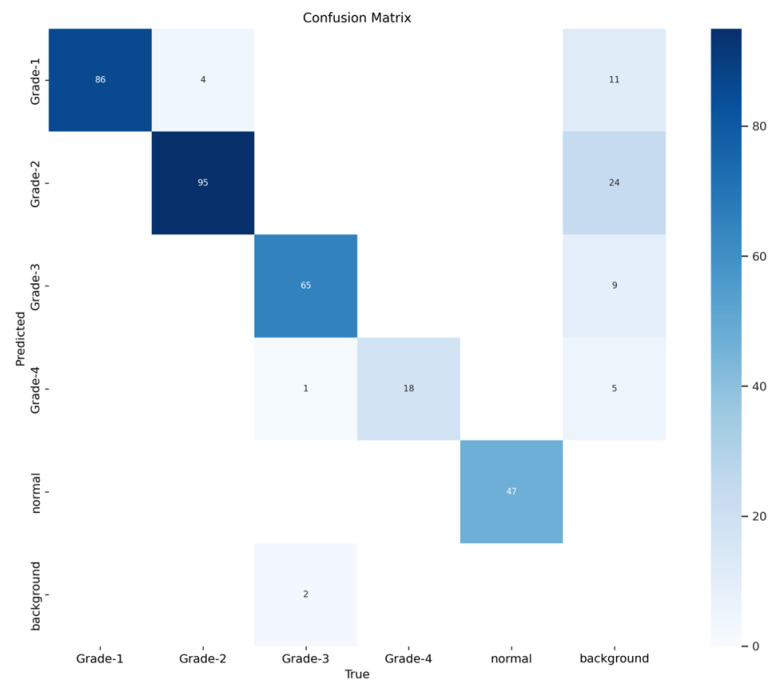
YOLOv8 validation confusion matrix.

**Figure 17:**
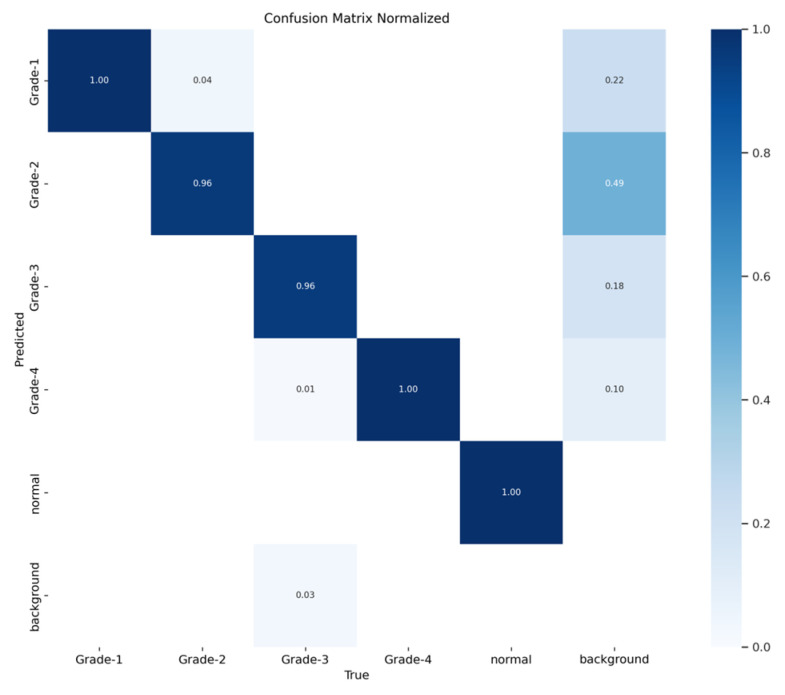
YOLOv8 validation confusion matrix normalized.

**Figure 18:**
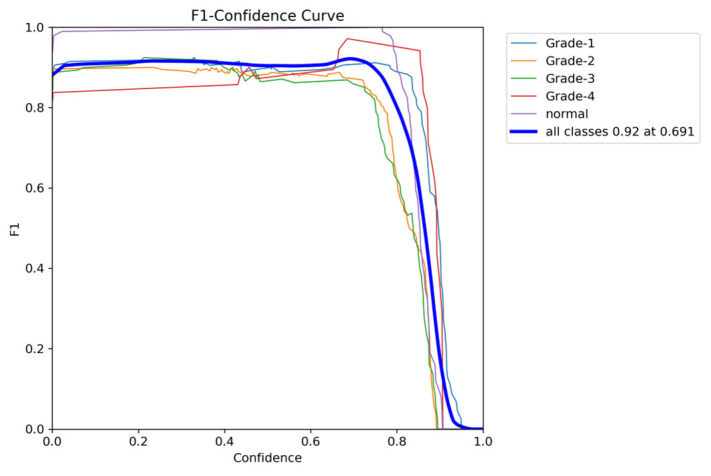
The validation F1-score and confidence curve.

**Figure 19:**
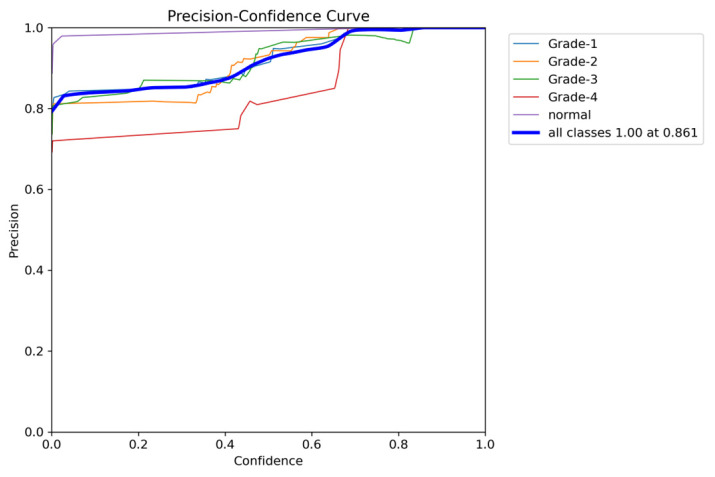
The validation precision and confidence curve.

**Figure 20:**
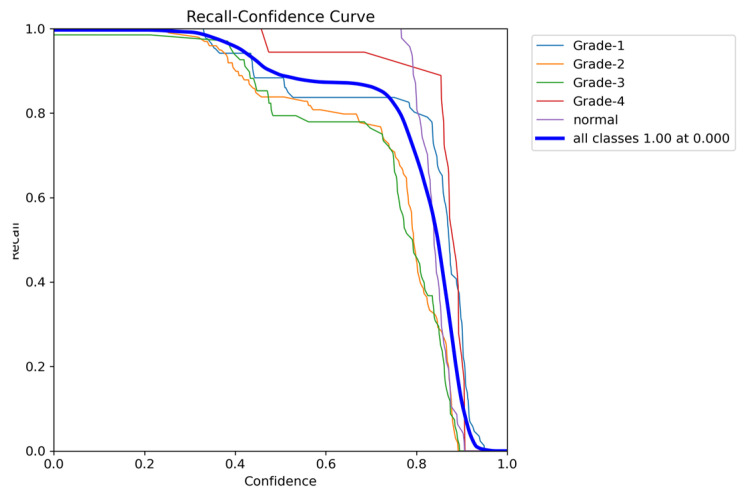
The validation recall and confidence curve.

**Figure 21:**
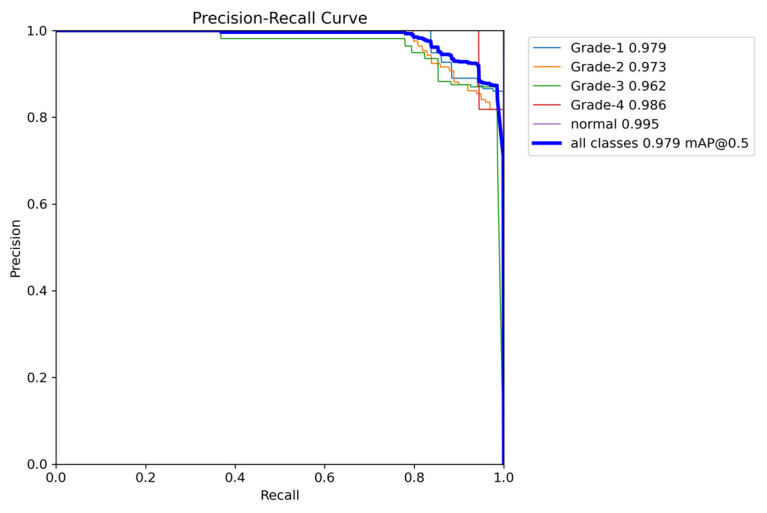
The validation precision and recall curve.

Figure 16 demonstrates the validation confusion matrices for YOLO version 8. The confusion matrices illustrate the classification performance of each model, providing insights into false positives and false negatives that may have significant clinical implications. Figure 17 demonstrates the validation confusion matrices for YOLO version 8 after dataset normalization. Figure 18, Figure 19, Figure 20 and Figure 21 demonstrate the validation curve.

For a comparative analysis of GMH detection capabilities, Table 6 summarizes the performance metrics of YOLOv8 against ResNet-18, ResNet-50, and ResNet-152, providing a comprehensive overview of each model’s precision, recall, and mean average precision. This comparison underscores YOLOv8’s superior mAP50 and mAP50-95 values, illustrating its efficacy in detecting and classifying GMH cases.

## 5. Model Evaluation

The addition of class weights and k-fold cross-validation resulted in significant improvements in model recall, as shown in Table 7, particularly for underrepresented classes. Notably, the recall for Grade 1 and Grade 2 increased substantially, indicating the model’s enhanced ability to consistently detect positive instances from these classes. However, this improvement in recall came with a slight trade-off in precision, which impacted the mean average precision (mAP), particularly under the more rigorous mAP50-95 metric. This trade-off suggests that while the model has become more adept at handling imbalanced data, its precision in distinguishing between similar or overlapping classes has slightly diminished. Despite this, the inclusion of these techniques enhanced the model’s robustness and generalizability across various subsets of data, making it more reliable in real-world scenarios.

To comprehensively evaluate the model’s performance, several key metrics were analyzed: the F1–Confidence curve, Precision–Recall (PR) curve, and Recall–Confidence curve.

**Figure 22:**
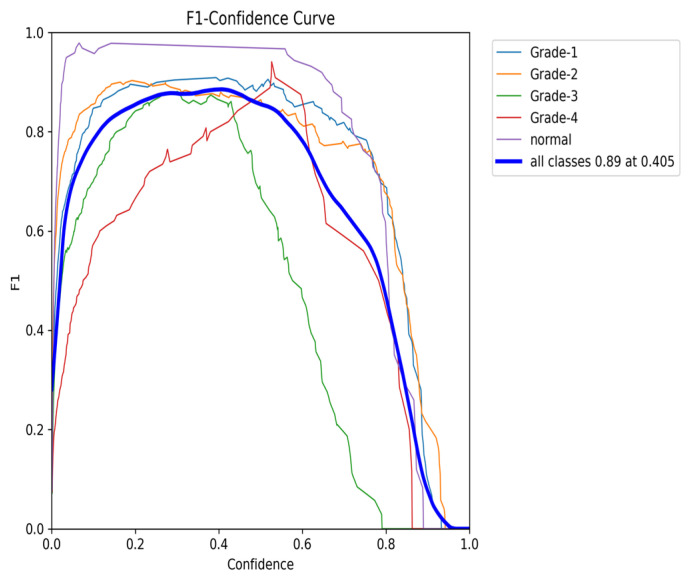
The validation F1-score and confidence curve after adding weights and K-folds.

The F1–Confidence curve (Figure 22) demonstrates how the F1-score varies with the confidence threshold across different classes. The model achieves an optimal F1-score of 0.89 at a confidence threshold of 0.405 for the combined class output, indicating strong performance across multiple categories. However, classes like Grade 3 exhibit a steeper decline in F1-score as the confidence threshold increases, highlighting the model’s sensitivity to confidence variations for certain classes.

**Figure 23:**
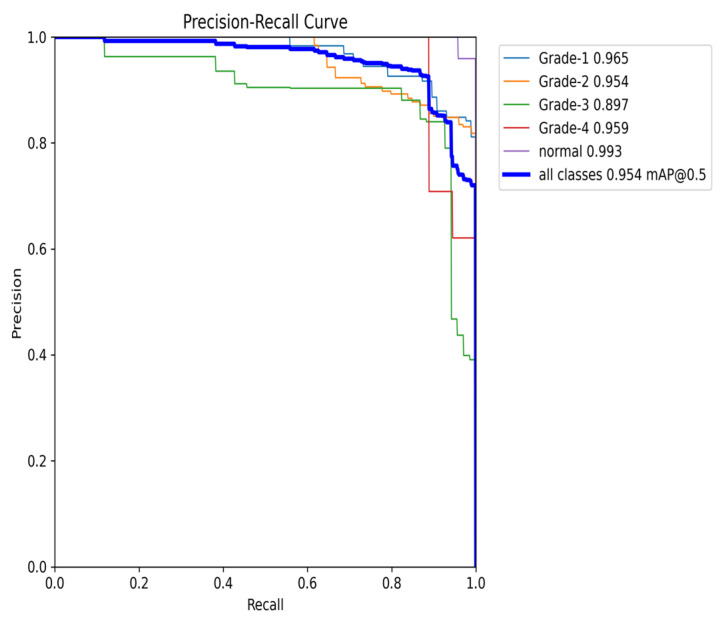
The validation precision and recall curve after adding weights and K-fold.

The Precision–Recall (PR) curve (Figure 23) provides further insights into the balance between precision and recall. At an IoU threshold of 0.5, the model achieves an overall mean average precision (mAP) of 0.954. While classes such as Grade 1 and Grade 4 maintain high precision, the slight decline in precision, particularly for Grade 2, aligns with the trade-off between recall and precision. Nonetheless, the PR curve demonstrates the model’s ability to maintain high precision, even when balancing recall, making it especially effective in detecting classes with fewer instances.

**Figure 24:**
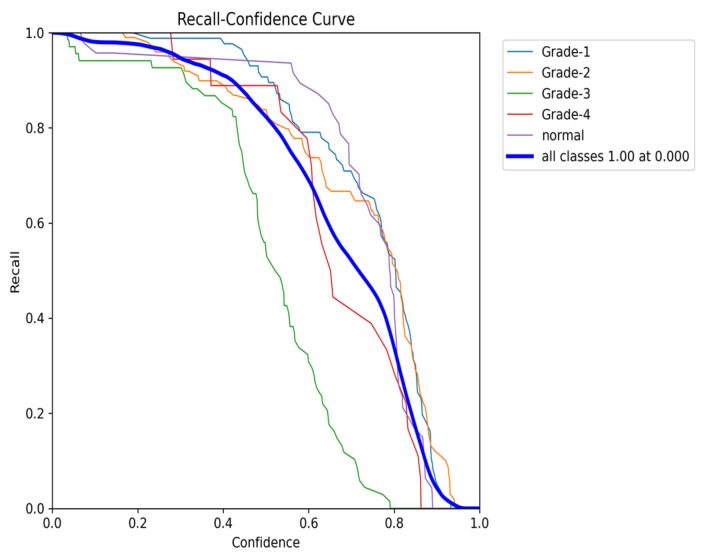
The validation recall and confidence curve after adding weights and K-fold.

The Recall–Confidence curve (Figure 24) highlights how recall varies across confidence levels. For the combined class output, the model achieves a perfect recall of 1.0 at a confidence threshold of 0, though recall values for certain classes, such as Grade 3, exhibit sharper declines as the confidence threshold increases. This behavior illustrates the model’s strength in maintaining high recall values, particularly for underrepresented classes, despite some loss at higher confidence thresholds.

**Figure 25:**
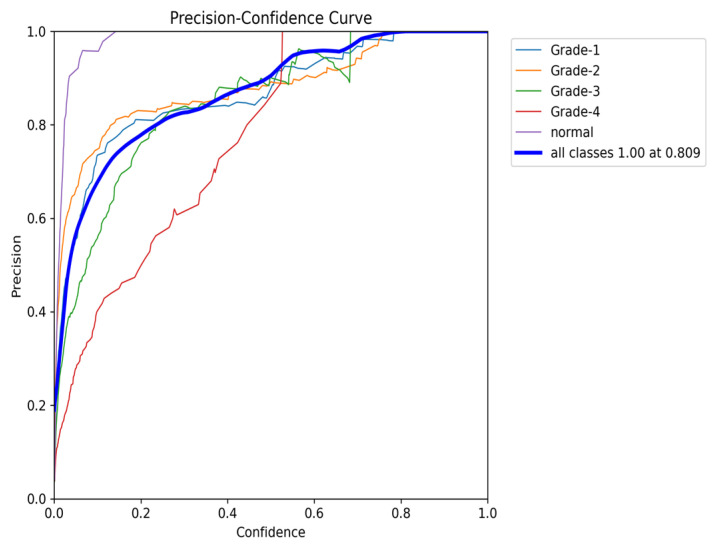
The validation precision and confidence curve after adding weights and K-fold.

Finally, the Precision–Confidence curve (Figure 25) further emphasizes the trade-off between precision and confidence. The model maintains high precision at lower confidence thresholds, but precision for classes like Grade 3 and Grade 4 drops off as the confidence level rises. This pattern reflects the overall behavior observed throughout the evaluation, where a high precision at lower thresholds is balanced by a reduction in performance for certain classes at higher confidence levels.

In summary, the evaluation metrics confirm that while the model exhibits some trade-offs between precision and recall, it remains highly robust and generalizable. The addition of class weights and k-fold cross-validation has improved its performance, particularly for imbalanced classes, with overall strong precision, recall, and F1-scores across varying confidence thresholds.

## 6. Discussion

**Figure 26:**
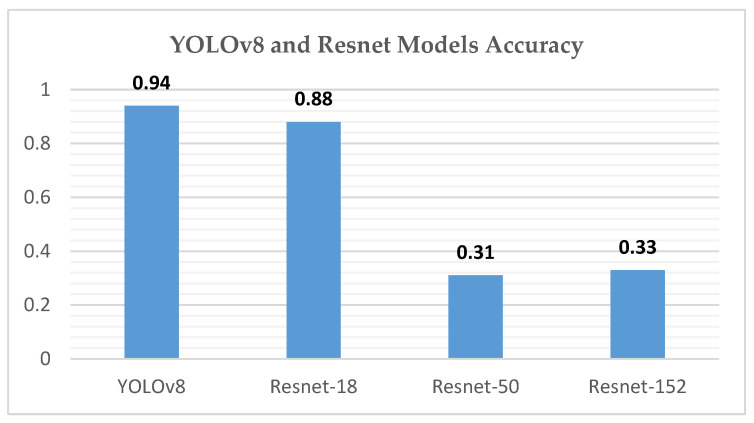
YOLOv8 and Resnet model accuracy chart.

**Figure 27:**
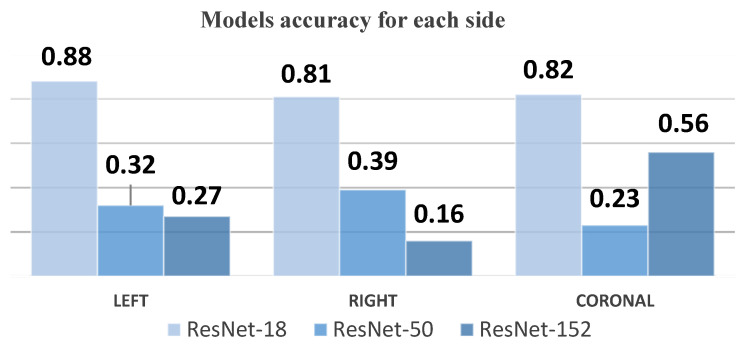
Model accuracy for each side chart.

Our study demonstrates the efficacy of the YOLOv8 model in the automated grading of GMH from cranial ultrasound images, achieving high precision and recall rates, particularly for the Normal and Grade 4 categories. The initial experiments using ResNet-18, ResNet-50, and ResNet-152 models provided critical insights into model performance and limitations. As shown in Figure 26, ResNet-18 showed promising results initially but failed to maintain accuracy across the entire dataset, particularly in handling complex hemorrhages like Grade 4 (mAP50 of 0.815). ResNet-50 struggled significantly with imbalanced data, particularly underrepresented classes like Grade 4, resulting in unsatisfactory outcomes (mAP50-95 of 0.670). ResNet-152 improved performance for coronal images but still performed inadequately for left- and right-side images (mAP50-95 of 0.690), as illustrated in Figure 27. These challenges led us to adopt the YOLOv8 model, which, through effective data augmentation and pre-processing, handled image variability better and improved performance on underrepresented classes (mAP50 of 0.979). However, the model faced challenges in distinguishing between Grade 2 and Grade 3 hemorrhages due to their similar bleeding patterns, highlighting the complexity of medical image classification. Despite this, YOLOv8’s strong performance indicates its potential as a reliable tool for radiologists, enhancing diagnostic accuracy and efficiency. Ensemble methods were considered as a potential alternative to improve classification performance, combining the strengths of YOLOv8 with ResNet models. However, these methods did not yield significant improvements compared to YOLOv8’s single model performance. Future research should focus on refining feature extraction and classification strategies to address closely related classes and explore integrating additional clinical data to further improve diagnostic capabilities.

### 6.1. Challenges and Limitations

Throughout this study, we encountered several significant challenges, primarily related to data availability and quality. The most prominent challenge was the limited size of the dataset, which impacted the model’s performance. When trained on this small dataset, the models tended to overfit, showing strong results on the training data but failing to generalize effectively to new, unseen cases. This limitation also affected the models’ accuracy, as they struggled to learn meaningful patterns from the limited data available.

To mitigate these issues, we implemented data augmentation techniques that expanded the size and diversity of the dataset. This approach improved the model’s ability to generalize by simulating variations in the data, thus reducing overfitting and enhancing overall performance.

Another key challenge was the quality of the initial images. The cranial ultrasound scans we received from the hospital were often unclear or of low resolution, making it difficult for models such as ResNet-18, ResNet-50, and ResNet-152 to accurately detect and classify hemorrhage locations. To address this, we employed a more rigorous data annotation process, ensuring that the images were precisely labeled. This improvement in data quality allowed the YOLOv8 model to perform significantly better, leading to more accurate and reliable detection and classification of hemorrhage sites.

Despite these challenges, the steps taken to address data limitations and improve image quality have led to a more robust model. However, we acknowledge that the limited dataset and lack of external validation remain constraints, which we aim to address in future work through collaboration with other institutions and exploring advanced image enhancement techniques.

### 6.2. Generalizability and External Validation

We acknowledge the absence of external validation in this study, as the model was trained and tested exclusively on data from King Fahad Hospital. While we recognize the importance of external datasets for assessing generalizability, the availability of neonatal ultrasound data is extremely limited. Due to privacy regulations and ethical considerations, we were unable to acquire data from other hospitals. Furthermore, there are no publicly available datasets of neonatal cranial ultrasound images, particularly for conditions as rare as germinal matrix hemorrhage (GMH).

Despite this limitation, our dataset remains valuable due to its high-quality imaging and detailed labeling of a rare condition. Each image was rigorously selected and annotated, ensuring that the model was trained on data of exceptional quality. Additionally, the use of advanced data augmentation techniques enhances the model’s ability to generalize by simulating variations that could occur in different clinical settings.

While external validation is not feasible at this stage, we believe that the model provides a strong foundation for GMH detection and classification in neonates. In future work, we aim to collaborate with other institutions to explore the possibility of data sharing, adhering to privacy and ethical standards, and eventually validating the model on diverse datasets.

Given the rarity of neonatal cranial ultrasound data and the constraints of patient privacy, this study represents a significant step forward in GMH research, with the potential to inform future work on this critical condition.

## 7. Conclusions

In conclusion, this study demonstrates the efficiency of the YOLOv8 model in the automated detection and classification of Germinal Matrix Hemorrhage (GMH) in premature neonates using cranial ultrasound imaging. Employing advanced deep learning techniques, including transfer learning and data augmentation, the model analyzed images from a dataset of 586 patients, categorizing them into five distinct grades. The YOLOv8 model achieved a mean average precision (mAP50) of 0.979 and a mAP50-95 of 0.724, highlighting its capability to distinguish between various GMH grades with high accuracy. Despite challenges in differentiating between closely similar grades, the model’s overall performance supports its potential as a substantial aid in clinical settings, enhancing the accuracy and efficiency of GMH diagnosis. This could significantly impact patient management by facilitating early intervention and potentially improving neurodevelopmental outcomes in this vulnerable population.

## Figures and Tables

**Table 1:** Resnet-18 accuracy on each side.

Classifier	Side	Accuracy
Resnet-18	Left	0.88
	Right	0.81
	Coronal	0.82

**Table 2:** Resnet-50 evaluation on each side.

Classifier	Side	Accuracy	Recall	Precision	F1-Score	Support
Resnet-50	Left	0.32	0.32	0.16	0.21	81
	Right	0.39	0.39	0.37	0.29	104
	Coronal	0.23	0.23	0.27	0.21	135

**Table 3:** Resnet-150 evaluation on each side.

Classifier	Side	Accuracy	Recall	Precision	F1-Score	Support
Resnet-152	Left	0.27	0.27	0.28	0.20	81
	Right	0.16	0.16	0.10	0.09	104
	Coronal	0.56	0.56	0.56	0.54	135

**Table 4:** Model accuracy on each side.

Model	Left	Right	Coronal
ResNet-18	0.88	0.81	0.82
ResNet-50	0.32	0.39	0.23
ResNet-152	0.27	0.16	0.56

**Table 5:** Validation metrics of YOLOV8 in our experiment.

Class	Images	Box Precision (P)	Recall (R)	mAP50	mAP50-95
All Classes	318	0.994	0.866	0.979	0.724
Grade 1	318	0.989	0.837	0.979	0.798
Grade 2	318	1	0.0.774	0.973	0.707
Grade 3	318	0.981	0.774	0.962	0.639
Grade 4	318	1	0.943	0.986	0.723
Normal	318	0.998	1	0.995	0.753

**Table 6:** Comparative performance of YOLOv8, ResNet-18, ResNet-50, and ResNet-152 on GMH Detection.

Model	Precision (P)	Recall (R)	mAP50	mAP50-95
**YOLOv8**	0.861	1.00	0.979	0.724
**Resnet-18**	0.812	0.889	0.815	0.651
**Resnet-50**	0.831	0.900	0.842	0.670
**Resnet-152**	0.845	0.923	0.865	0.690

**Table 7:** Comparative performance of YOLOv8 original results and after adding weights and K-fold.

Metric	Original Results	With Weights and K-Fold
**Box Precision (P)**	0.994	0.861
**Recall (R)**	0.866	0.913
**mAP50**	0.979	0.954
**mAP50-95**	0.724	0.619

## Data Availability

Data were obtained from King Fahd Hospital of Imam Abdulrahman bin Faisal University and are available from the authors with the permission of King Fahd Hospital of the University.
